# Effects of a Two-Strain *Lactobacillus* Formulation (*Lactobacillus rhamnosus* L34 and *Lactobacillus paracasei* B13) on Inflammatory and Oxidative Markers, Epithelial Junction Proteins, and Fecal Microbiota in Rats with DNBS-Induced Colitis

**DOI:** 10.3390/ijms27219961

**Published:** 2026-11-06

**Authors:** Pakkapon Rattanachaisit, Nathawadee Lerttanatum, Somying Tumwasorn, Natthaya Chuaypen, Thasinas Dissayabutra, Duangporn Werawatganon

**Affiliations:** 1 Center of Excellence in Alternative and Complementary Medicine for Gastrointestinal and Liver Diseases, Chulalongkorn University, Bangkok 10330, Thailand; pakkapon.r@chula.ac.th; 2 Department of Physiology, Faculty of Medicine, Chulalongkorn University, Bangkok 10330, Thailand; 3 Department of Pathology, Faculty of Medicine, Chulalongkorn University, Bangkok 10330, Thailand; 4 Department of Microbiology, Faculty of Medicine, Chulalongkorn University, Bangkok 10330, Thailand; 5 Center of Excellence in Hepatitis and Liver Cancer, Chulalongkorn University, Bangkok 10330, Thailand; 6 Department of Biochemistry, Faculty of Medicine, Chulalongkorn University, Bangkok 10330, Thailand

**Keywords:** *Lactobacillus*, probiotics, colitis, oxidative stress, epithelial barrier, fecal microbiota

## Abstract

Inflammatory bowel disease (IBD) involves dysregulated mucosal immunity, oxidative stress, impaired epithelial barrier integrity and gut microbial dysbiosis. We evaluated a two-strain formulation of *Lactobacillus rhamnosus* L34 and *Lactobacillus paracasei* B13 in DNBS-induced colitis. Twenty-four male Sprague Dawley rats were randomised into three groups (*n* = 8): control, acute colitis (AC; DNBS 35 mg/0.25 mL per rat rectally on days 0 and 1), and probiotic treated (RP; 3.6 × 10^9^ CFU daily by gavage, days −3 to 6). DNBS raised Ameho histological scores, serum TNF-α, colonic MDA and MPO positivity and lowered IL-10, occludin and claudin-1 (all *p* < 0.05). Relative to AC, probiotic-treated rats showed lower TNF-α (*p* = 0.002) and MPO positivity (*p* < 0.001) and higher IL-10 (*p* = 0.001), occludin (*p* = 0.014) and claudin-1 (*p* = 0.001). Colonic MDA and Ameho scores were numerically lower in the RP group but these differences did not reach statistical significance (*p* = 0.085 and *p* = 0.310). Serum IL-17A was unchanged. Microbial community composition differed among groups (PERMANOVA pseudo-F = 5.798, R^2^ = 0.356, *p* = 0.0001), with the probiotic-associated community distinct from both control and colitis communities; distance-based analysis detected no convergence toward the control community. The formulation attenuated inflammatory and barrier outcomes and altered microbiota composition without restoring it.

## 1. Introduction

Inflammatory bowel disease (IBD) is an idiopathic chronic gastrointestinal disease and the term mainly refers to two conditions: ulcerative colitis (UC) and Crohn’s disease (CD). It is characterized by chronic and spontaneously relapsing inflammation due to a complex interaction of genetic, microbial, and environmental factors, which results in continuous activation of the mucosal immune system [1]. The pathophysiologic processes of IBD include migration of inflammatory cells such as neutrophils, basophils, and other leukocytes to the mucosa, and secretion of cytokines, arachidonic acid metabolites, and free radicals results in oxidative damage to intestinal tissue [2,3].

Changes in intestinal microbiota composition and function—collectively termed dysbiosis—have been linked to the pathogenesis of IBD, as supported by both experimental and clinical evidence [4]. The commensal microbial flora promotes immune system maturation and confers the ability to detect and eliminate pathogenic microorganisms within the intestinal lumen. It also contributes to colonic morphology, regulation of the inflammatory response, and maintenance of the colonic mucosal barrier, collectively preventing entry of pathogens and allergens [5]. Under normal conditions, host microbiota homeostasis is maintained in a compositional equilibrium. When this balance is disrupted, intestinal microflora diversity declines, facilitating expansion of pathogenic bacteria [6]. The contribution of bacteria to intestinal inflammation is supported by the clinical observation that remission can be achieved with antibiotics such as metronidazole or ciprofloxacin [7] or the failure of inflammation induction in germ-free animals [8].

Probiotics are microorganisms that help to restore and maintain microbial balance in cases of dysbiosis. They provide health benefits in various pathways, including modulation of intestinal bowel flora, modulation of immune response, and enhancing intestinal barrier function [9,10]. Probiotics can modulate the intestinal microbiota by producing competitive metabolic interactions with pathogens, production of anti-microbial peptides, or inhibition of epithelial adherence and translocation by pathogens [11].

Given the pivotal role of the gut microbiota in the pathogenesis of IBD, microbiota-targeted therapies have been extensively investigated as potential treatment strategies, among which probiotics represent one of the most promising alternatives. Appropriate dosing and strain composition of probiotics have been shown to exert beneficial effects in both animal models and human studies. Notably, probiotics have demonstrated positive effects in both the induction and maintenance of remission in patients with CD and UC. Among the most extensively studied probiotic organisms are *Lactobacillus* and *Bifidobacterium* species. For instance, *Lactobacillus plantarum*, *Lactobacillus paracasei*, *Saccharomyces boulardii*, and *Bifidobacterium infantis* have all demonstrated the ability to alleviate intestinal inflammation and improve disease symptoms in both murine models and patients with enteritis [12,13].

*L. rhamnosus* L34 and *L. paracasei* B13 can be readily cultivated in our laboratory and have previously demonstrated anti-inflammatory properties, as well as the ability to reduce intestinal permeability in several disease models, including alcoholic hepatitis [14], sepsis [15], *Clostridioides difficile* infection [16], non-alcoholic steatohepatitis [17], and acute pancreatitis [18]. Given these beneficial effects in conditions characterized by inflammation and impaired gut barrier function, we sought to investigate the potential therapeutic effects of these *Lactobacillus* strains in an IBD model.

Combinations of probiotic strains have been reported to confer greater benefit than single-strain preparations in some models, partly through enhanced induction of anti-inflammatory cytokines [19], and this provided the rationale for evaluating a combined preparation in the present work. We therefore examined whether a formulation combining *L. rhamnosus* L34 and *L. paracasei* B13 would attenuate the severity of DNBS-induced colitis in rats, assessed by inflammatory mediators, oxidative stress markers, epithelial junction protein expression, histopathology and faecal microbiota composition. Because the formulation was tested only as a two-strain combination, the present design evaluates the effect of the combined preparation as administered; it cannot attribute effects to either strain individually, nor determine whether the strains interact additively or synergistically. Such questions require a factorial design incorporating single-strain arms and are a priority for future work. The findings may nonetheless inform the potential role of probiotic intervention as an adjunctive strategy in IBD.

## 2. Results

### 2.1. Effects of Probiotics on Colon Histopathology

**Figure 1:**
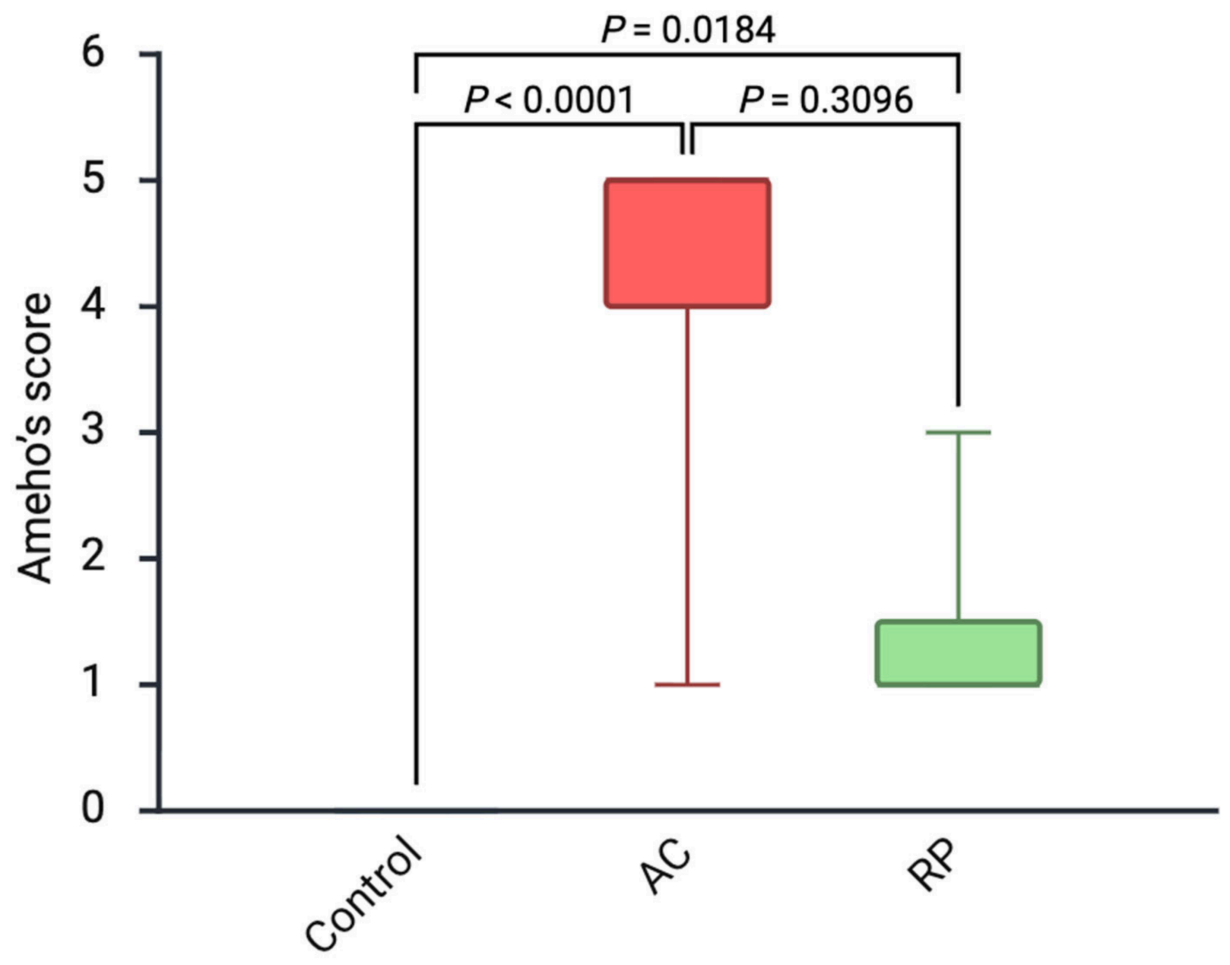
Effects of probiotic treatment on histological severity of colitis. Box plots showing Ameho’s histological scores in the control, AC and RP groups (*n* = 8 per group).

As shown in Figure 1, Ameho’s histological scores differed significantly among the groups (Kruskal–Wallis H = 19.51, *p* < 0.0001, ε^2^ = 0.834). The AC group exhibited markedly higher scores than the control group (5 [2–5] vs. 0 [0–0]; Hodges–Lehmann difference 5, 95% CI 1 to 5; adjusted *p* < 0.0001), indicating severe colonic mucosal injury. In the RP group, Ameho’s scores were numerically lower than those in the AC group (1 [1–2.5] vs. 5 [2–5]; Hodges–Lehmann difference −4, 95% CI −4 to 0; rank-biserial r = −0.688), but this difference did not reach statistical significance (adjusted *p* = 0.3096). Ameho’s scores in the RP group remained significantly higher than those in the control group (1 [1–2.5] vs. 0 [0–0]; adjusted *p* = 0.0184).

**Figure 2:**
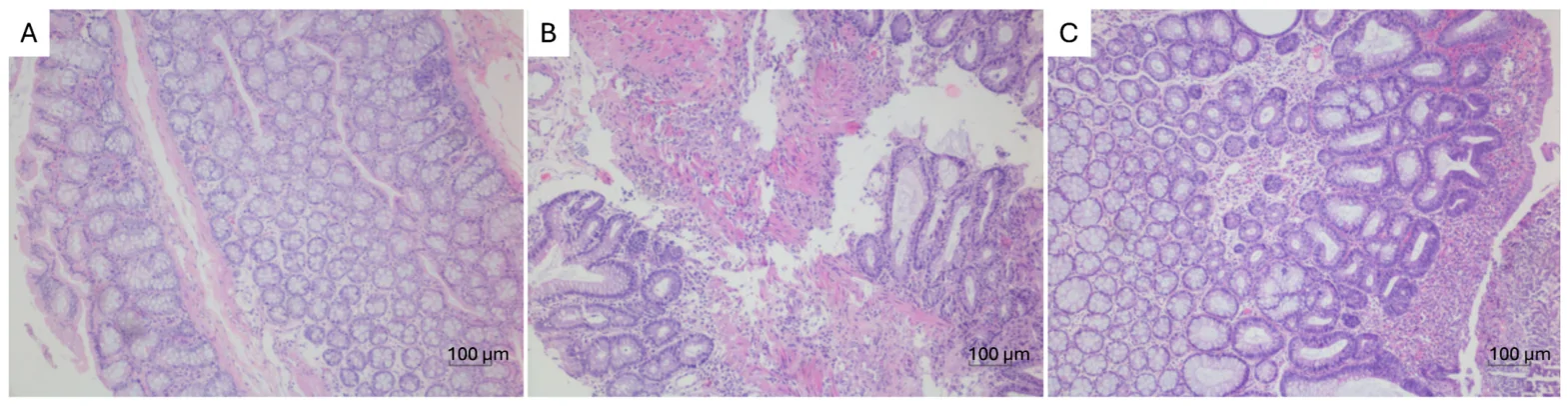
Representative colonic histopathology (H&E, 100×) in each group. (**A**) Control; (**B**) acute colitis; and (**C**) probiotic treated. Scale bar 100 µm.

Histopathological examination supported these findings. The control group showed normal colonic architecture with intact epithelial lining and well-organized crypt structures. In contrast, the AC group demonstrated marked histopathological alterations, including epithelial disruption, inflammatory cell infiltration, mucosal ulceration, and loss of crypt architecture. In the RP group, histological damage appeared partially attenuated, with reduced inflammatory infiltration and improved mucosal structure compared with the AC group (Figure 2).

### 2.2. Effects of Probiotics on TNF-α, IL-10, and IL-17 Levels

**Figure 3:**
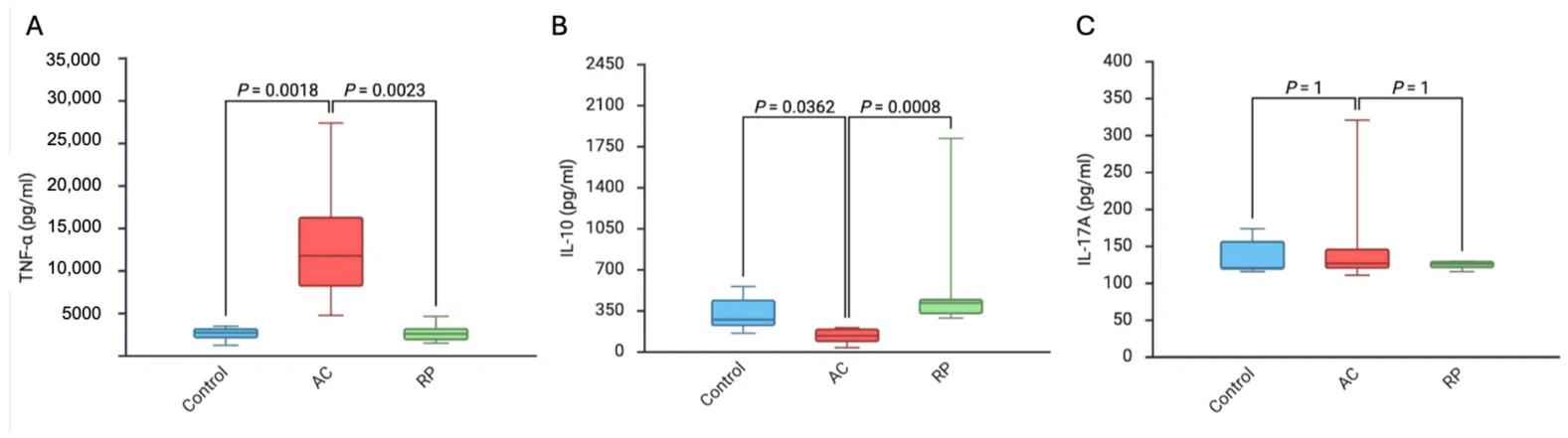
Effects of probiotic treatment on serum cytokine concentrations. Box plots showing serum TNF-α (**A**), IL-10 (**B**) and IL-17A (**C**) in the control, AC and RP groups (*n* = 8 per group).

Serum TNF-α varied markedly across the three groups (Kruskal–Wallis H = 15.37, *p* = 0.0005, ε^2^ = 0.636; Figure 3). Concentrations in colitic animals were roughly four times those of controls (11,800 [6922–16,625] vs. 2745 [1815–3305] pg/mL; Hodges–Lehmann difference 9260 pg/mL, 95% CI 3880 to 14,160; adjusted *p* = 0.0018). This elevation was abolished by probiotic pretreatment (2610 [1778–3458] pg/mL; Hodges–Lehmann difference versus AC −9235 pg/mL, 95% CI −14,240 to −4210; adjusted *p* = 0.0023), with a rank-biserial correlation of −1.000 indicating that every probiotic-treated animal had a lower value than every colitic animal. No statistically significant difference was detected between the RP and control groups (adjusted *p* = 1.000; this Bonferroni-adjusted value is truncated at unity and indicates the absence of a detectable difference rather than equivalence).

Serum IL-10 showed the opposite pattern (Kruskal–Wallis H = 13.90, *p* = 0.0010, ε^2^ = 0.566). Colitis roughly halved circulating IL-10 relative to controls (140 [83.3–198] vs. 277 [192–492] pg/mL; Hodges–Lehmann difference −161 pg/mL, 95% CI −351 to −52; adjusted *p* = 0.0362), whereas animals receiving the probiotic formulation reached 418 [316–460] pg/mL, exceeding the colitic group by a Hodges–Lehmann difference of 259 pg/mL (95% CI 187 to 367; rank-biserial r = +1.000; adjusted *p* = 0.0008). IL-10 in the RP group was not statistically separable from control values (adjusted *p* = 0.7737).

In contrast, serum IL-17A concentrations were comparable among the three groups, with no statistically significant differences (control 121 [119.25–158.75] pg/mL; AC 127 [119.00–173.25] pg/mL; RP 126 [119.25–129.00] pg/mL; Kruskal–Wallis H = 0.304, *p* = 0.859; all adjusted pairwise *p* = 1.000). The wider interquartile range in the AC group reflects one animal with a markedly higher value (321 pg/mL); this value was retained in all analyses, and because all comparisons were rank-based it did not affect the result, a sensitivity analysis excluding it leaving the conclusion unchanged (Kruskal–Wallis *p* = 0.998).

### 2.3. Effects of Probiotics on MDA Level

**Figure 4:**
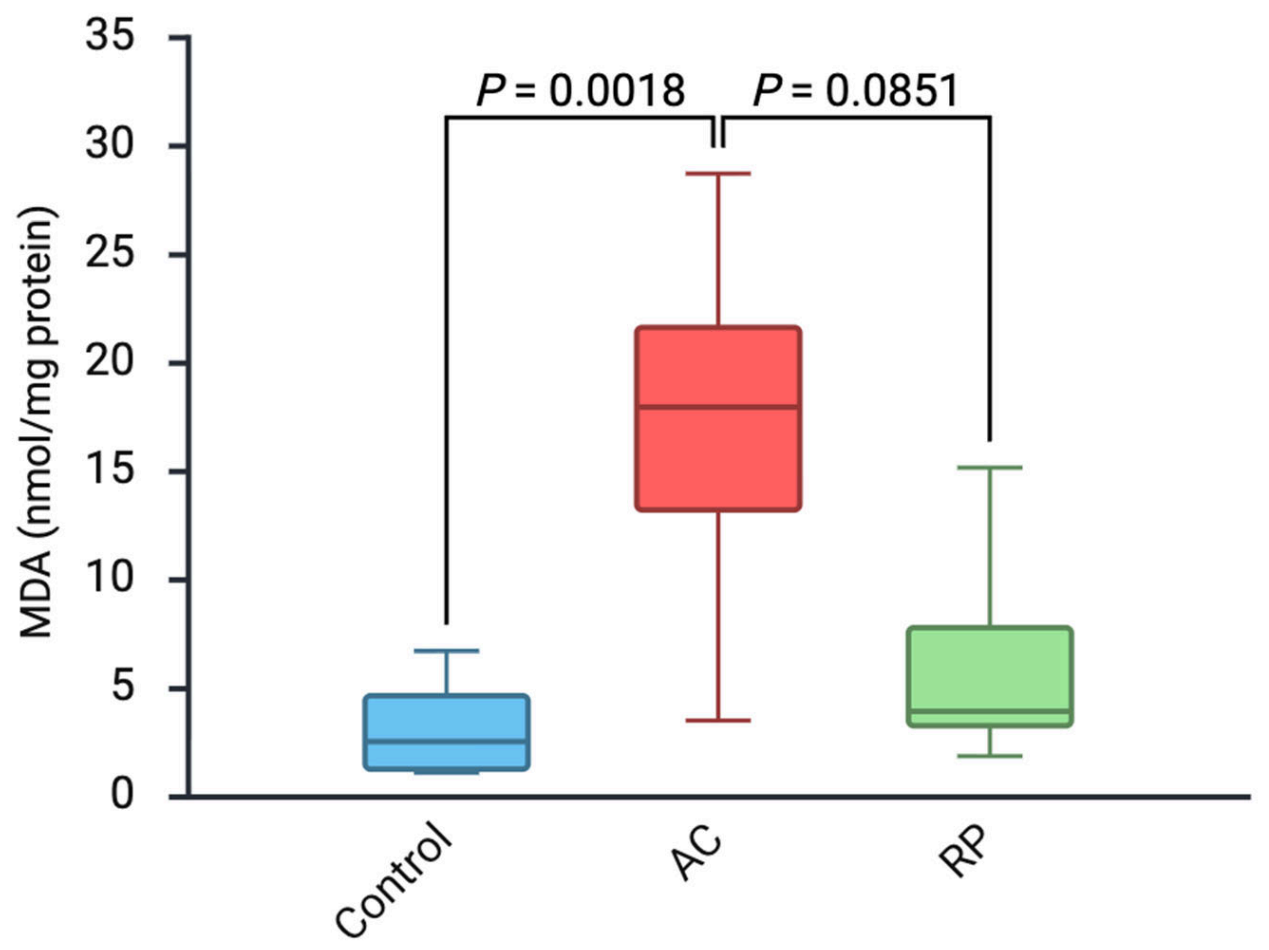
Effects of probiotics on colonic MDA. Box plots showing colonic malondialdehyde concentrations (nmol/mg protein) in the control, AC and RP groups (*n* = 8 per group).

Lipid peroxidation in colonic tissue followed the inflammatory pattern (Kruskal–Wallis H = 12.07, *p* = 0.0024, ε^2^ = 0.479; Figure 4). Colitic animals accumulated roughly sevenfold more MDA than controls (17.98 [11.50–23.84] vs. 2.56 [1.24–5.37] nmol/mg protein; Hodges–Lehmann difference 14.46 nmol/mg protein, 95% CI 8.37 to 20.60; adjusted *p* = 0.0018). Probiotic pretreatment was associated with substantially lower values (3.96 [2.79–8.89] nmol/mg protein; Hodges–Lehmann difference versus AC −11.94 nmol/mg protein, 95% CI −18.17 to −4.65; rank-biserial r = −0.750), although this comparison fell short of statistical significance (adjusted *p* = 0.0851). Colonic MDA in the RP group lay within the control range (adjusted *p* = 0.6478).

### 2.4. Effects of Probiotics on MPO Positivity

**Figure 5:**
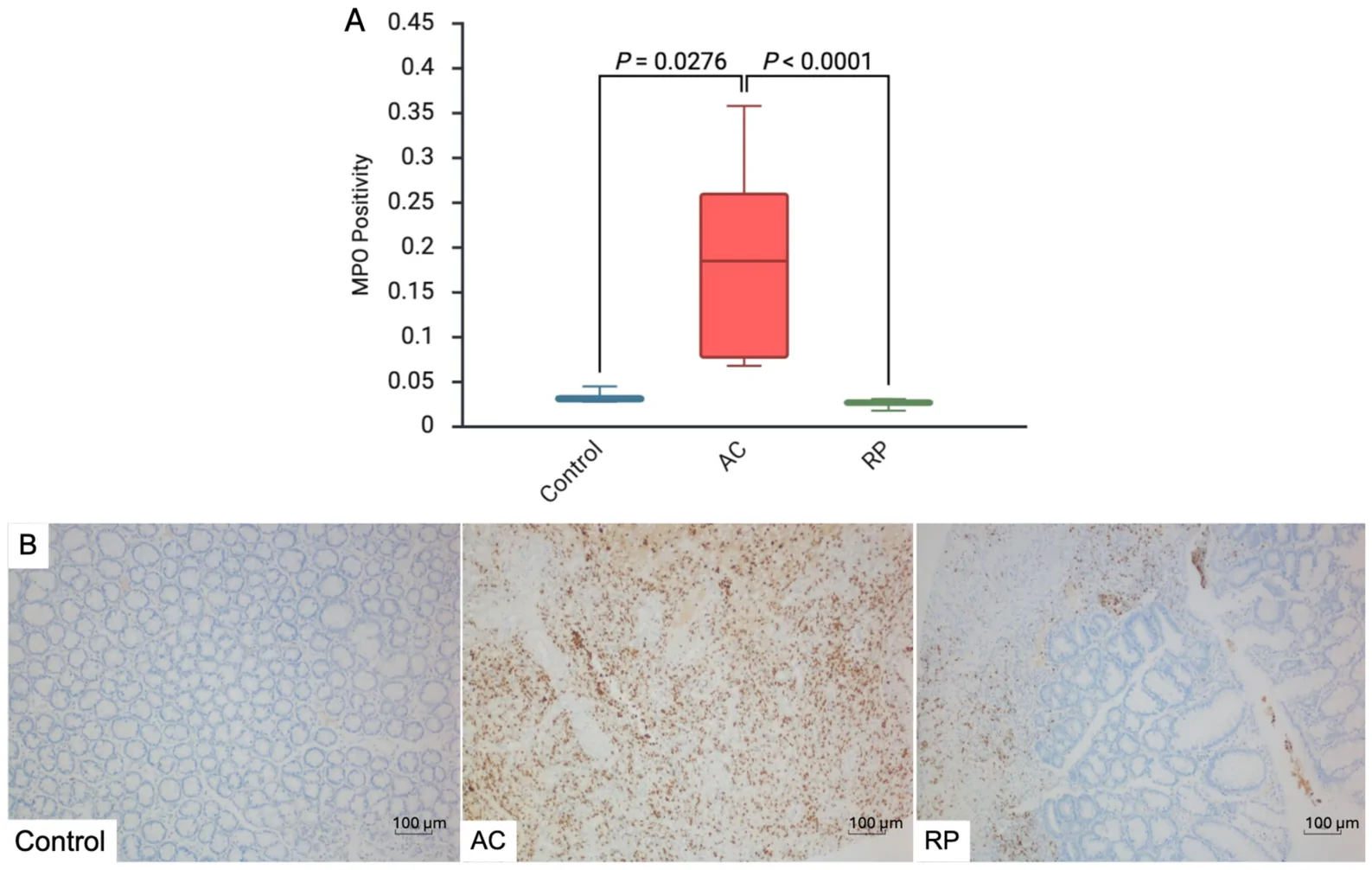
Effects of probiotics on colonic MPO positivity. (**A**) Box plots showing the MPO positive-pixel area fraction across groups (*n* = 8 per group). (**B**) Representative immunostained sections (100×; scale bar 100 µm).

Neutrophil infiltration, quantified as the MPO positive-pixel area fraction, was strongly affected by colitis induction (Kruskal–Wallis H = 17.97, *p* = 0.0001, ε^2^ = 0.760; Figure 5). The colitic group reached 0.1850 [0.0745–0.2653] against 0.0305 [0.0283–0.0373] in controls (Hodges–Lehmann difference 0.1535, 95% CI 0.0420 to 0.2360; adjusted *p* = 0.0276). Among probiotic-treated animals, MPO positivity fell to 0.0260 [0.0222–0.0290], a Hodges–Lehmann difference of −0.1590 relative to the colitic group (95% CI −0.2390 to −0.0480; rank-biserial r = −1.000; adjusted *p* < 0.0001). Control and RP values did not separate statistically (adjusted *p* = 0.3325).

### 2.5. Occludin and Claudin-1 Positivity

**Figure 6:**
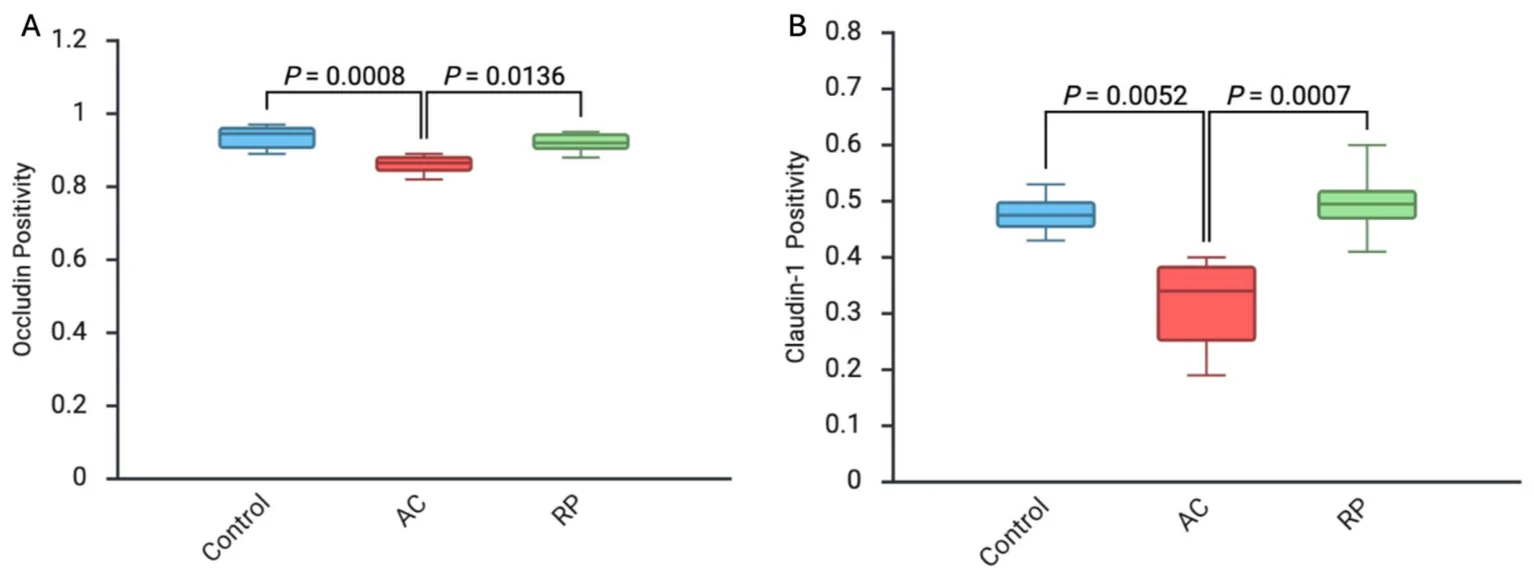

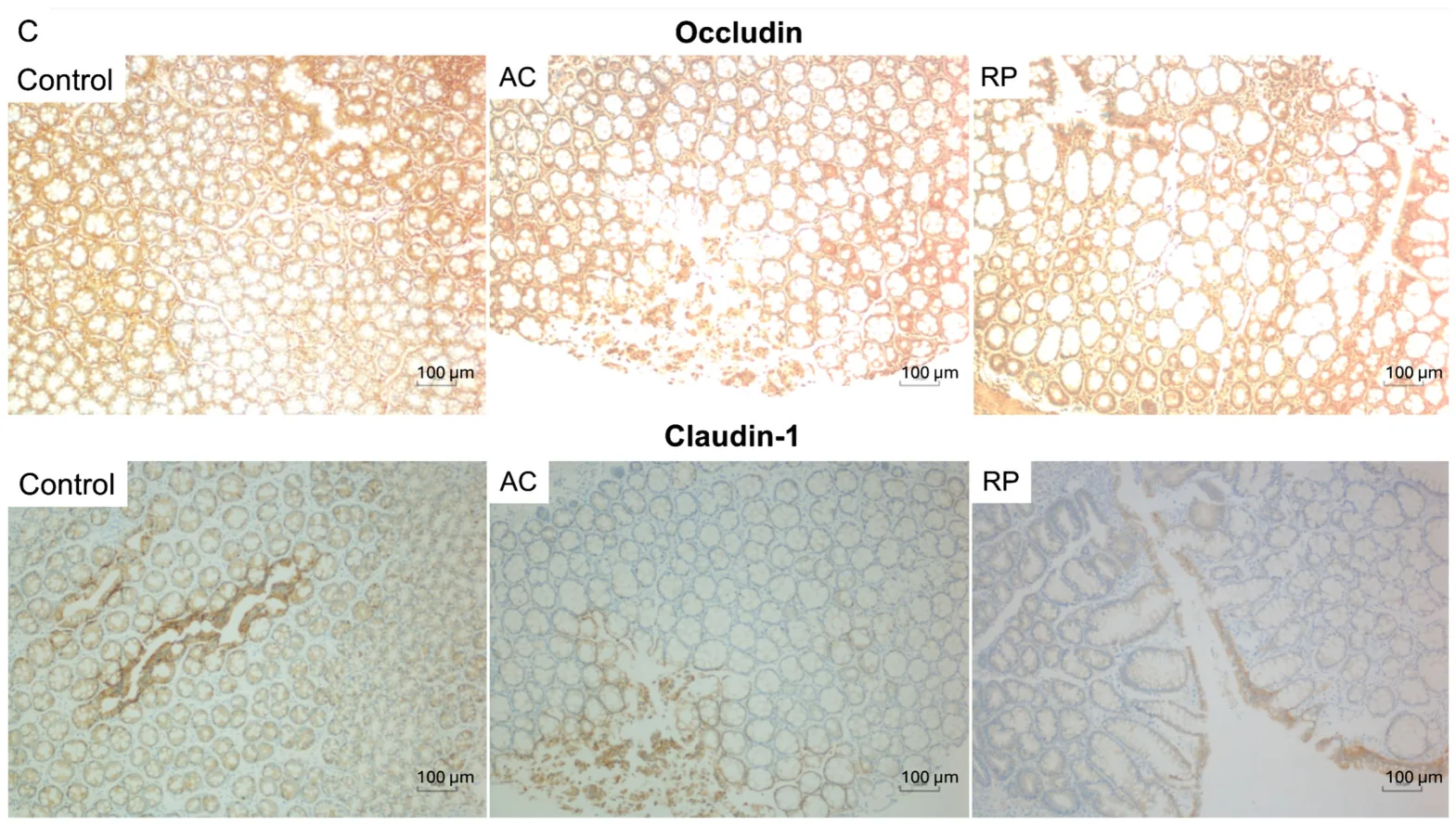
Effects of probiotics on occludin (**A**) and claudin-1 (**B**) positivity. Box plots showing the respective positive-pixel area fractions across groups (*n* = 8 per group); this measure is a dimensionless proportion between 0 and 1. (**C**) Representative immunostained sections (100×; scale bar 100 µm).

Epithelial junction proteins were likewise affected. Occludin positivity differed across groups (Kruskal–Wallis H = 14.72, *p* = 0.0006, ε^2^ = 0.606; Figure 6A), falling from 0.9450 [0.9025–0.9600] in controls to 0.8650 [0.8350–0.8800] after DNBS challenge (Hodges–Lehmann difference −0.0800, 95% CI −0.1100 to −0.0400; adjusted *p* = 0.0008). Animals given the probiotic formulation retained higher occludin positivity than colitic animals (0.9200 [0.8950–0.9475]; Hodges–Lehmann difference 0.0600, 95% CI 0.0300 to 0.0900; rank-biserial r = +0.922; adjusted *p* = 0.0136) and showed no statistically significant difference from controls (adjusted *p* = 1.000).

Claudin-1 behaved similarly (Kruskal–Wallis H = 15.69, *p* = 0.0004, ε^2^ = 0.652; Figure 6B). DNBS challenge reduced claudin-1 positivity from 0.4750 [0.4450–0.5125] to 0.3400 [0.2375–0.3875] (Hodges–Lehmann difference −0.1400, 95% CI −0.2400 to −0.0900; adjusted *p* = 0.0052), consistent with disrupted tight-junction integrity. In the RP group claudin-1 positivity reached 0.4950 [0.4700–0.5325], a gain over the colitic group of 0.1700 by Hodges–Lehmann estimation (95% CI 0.1000 to 0.2600; rank-biserial r = +1.000; adjusted *p* = 0.0007) and matching control values (adjusted *p* = 1.000).

### 2.6. Gut Microbiota

**Figure 7:**
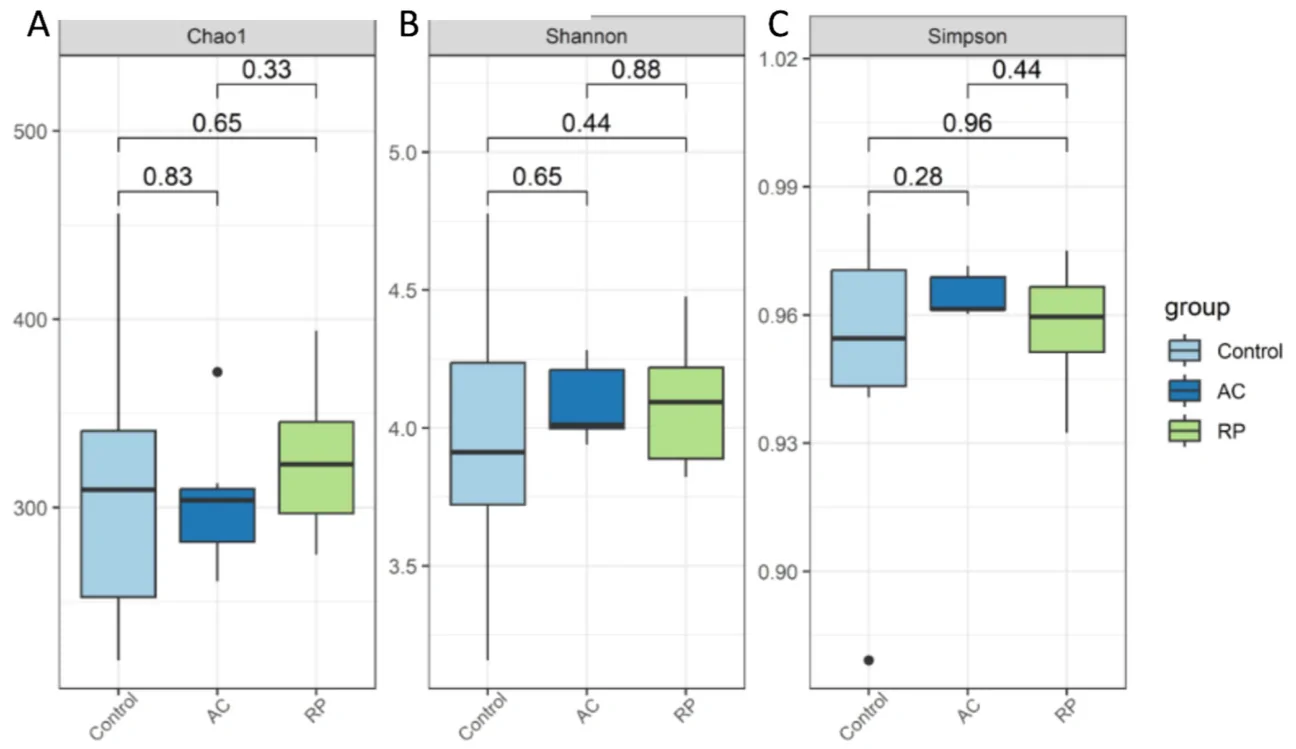
Effects of probiotic treatment on alpha diversity of faecal microbiota. Box plots showing (**A**) Chao1 richness, (**B**) Shannon diversity and (**C**) Simpson diversity in the control, acute colitis (AC) and probiotic-treated (RP) groups (*n* = 8 per group). Rarefaction and testing are described in Section 4.12.

**Figure 8:**
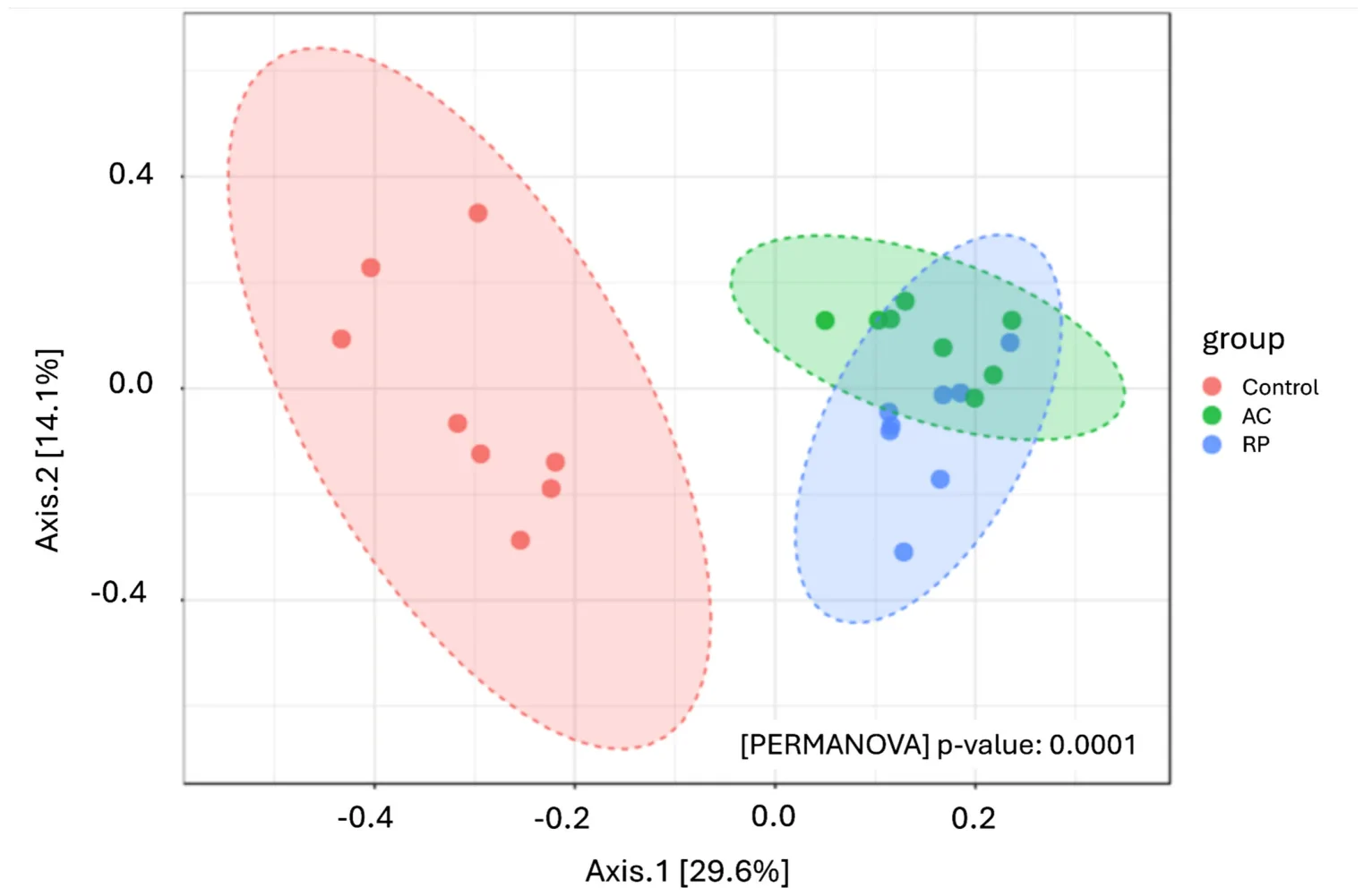
Effects of probiotic treatment on the beta diversity of faecal microbiota. Principal coordinate analysis (PCoA) plot based on Bray–Curtis dissimilarities illustrating overall microbial community composition in the control (red), AC (green) and RP (blue) groups (*n* = 8 per group). Each point represents one rat; ellipses indicate 95% confidence regions. Visual proximity in the ordination does not by itself indicate community convergence; see the distance-based analysis reported in the text.

No index of alpha diversity separated the three groups (Chao1 *p* = 0.655; Shannon *p* = 0.728; Simpson *p* = 0.500; observed ASVs *p* = 0.573; Kruskal–Wallis; Figure 7). Community structure, by contrast, differed substantially (PERMANOVA on Bray–Curtis dissimilarities: pseudo-F = 5.798, R^2^ = 0.356, *p* = 0.0001). Both challenged groups diverged from controls (Bonferroni-adjusted *p* = 0.0015 for AC and *p* = 0.0009 for RP), and the two challenged groups also differed from one another (adjusted *p* = 0.0030). Group dispersions were unequal (PERMDISP: F = 3.618, *p* = 0.041); within-group variability was lowest in the colitic group (mean distance to centroid 0.267, against 0.329 in RP and 0.369 in controls). Since the design was balanced, the PERMANOVA result remains interpretable, but it reflects dispersion as well as location. Applying the distance-based test described in Section 4.12, proximity to the control community did not differ significantly between the two challenged groups (AC 0.664 ± 0.028 versus RP 0.686 ± 0.028; Hodges–Lehmann difference −0.020, 95% CI −0.055 to +0.007; *p* = 0.141), and centroid separation from controls was virtually equal (AC 0.480, RP 0.473). The probiotic-associated community was therefore compositionally distinct from both comparison groups, with no sign of convergence toward the control state (Figure 8).

**Figure 9:**
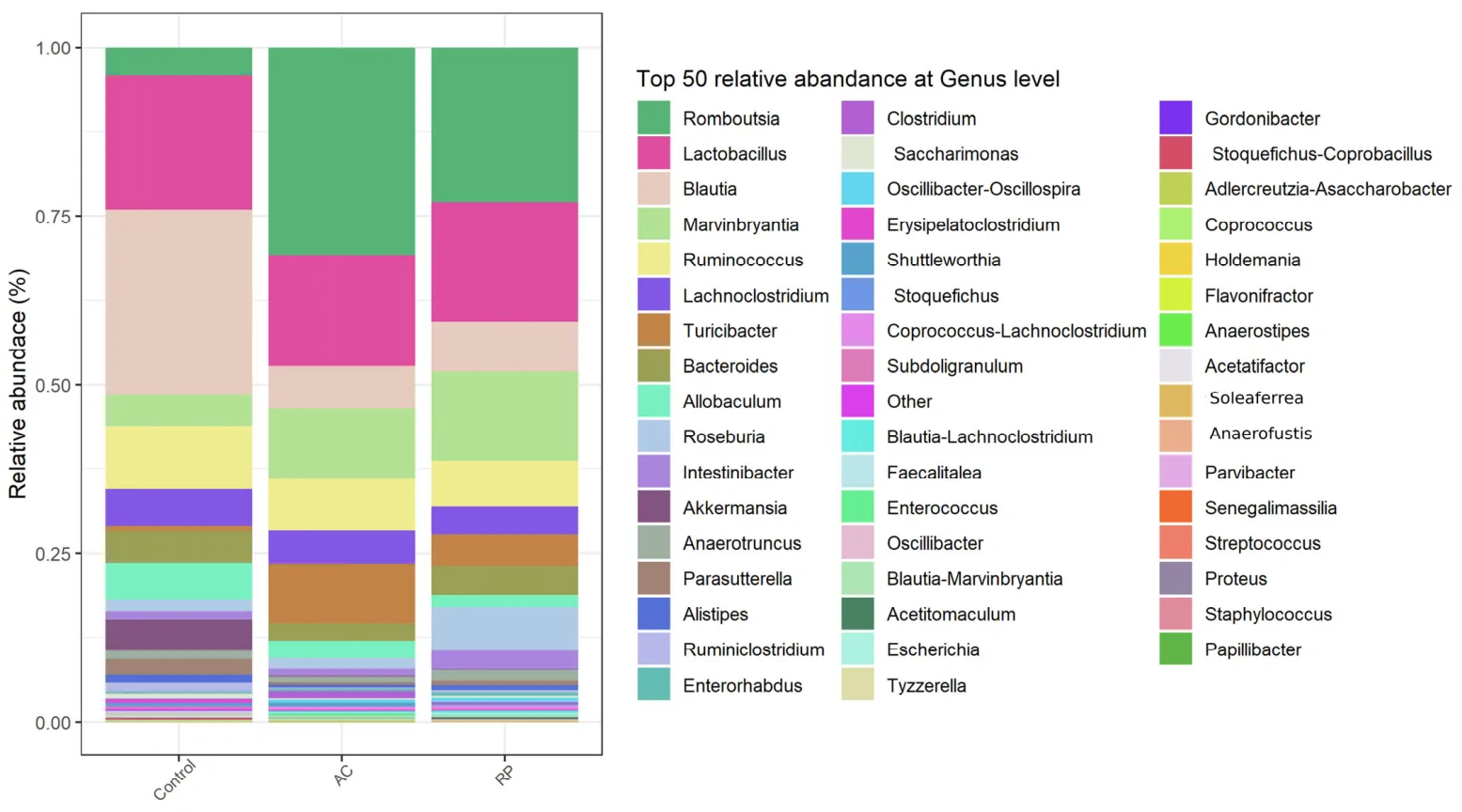
Relative abundance of gut microbiota at the genus level across groups. Stacked bar chart showing the 50 most abundant genera in the control, AC and RP groups (*n* = 8 per group).

Taxonomic profiling at the genus level showed that the AC group was characterised by a pronounced expansion of *Romboutsia* (median 19.96% versus 1.50% in Control) and *Turicibacter* (5.54% versus 0.17%), together with depletion of *Blautia* (3.34% versus 15.51%). In the RP group the expansion of *Romboutsia* (6.87%) and *Turicibacter* (2.25%) was substantially attenuated relative to the AC group, whereas *Blautia* remained low (2.25%). *Lactobacillus* relative abundance did not differ among groups (Control 9.19%, AC 9.35%, RP 10.18%; Kruskal–Wallis *p* = 0.833) (Figure 9).

**Figure 10:**
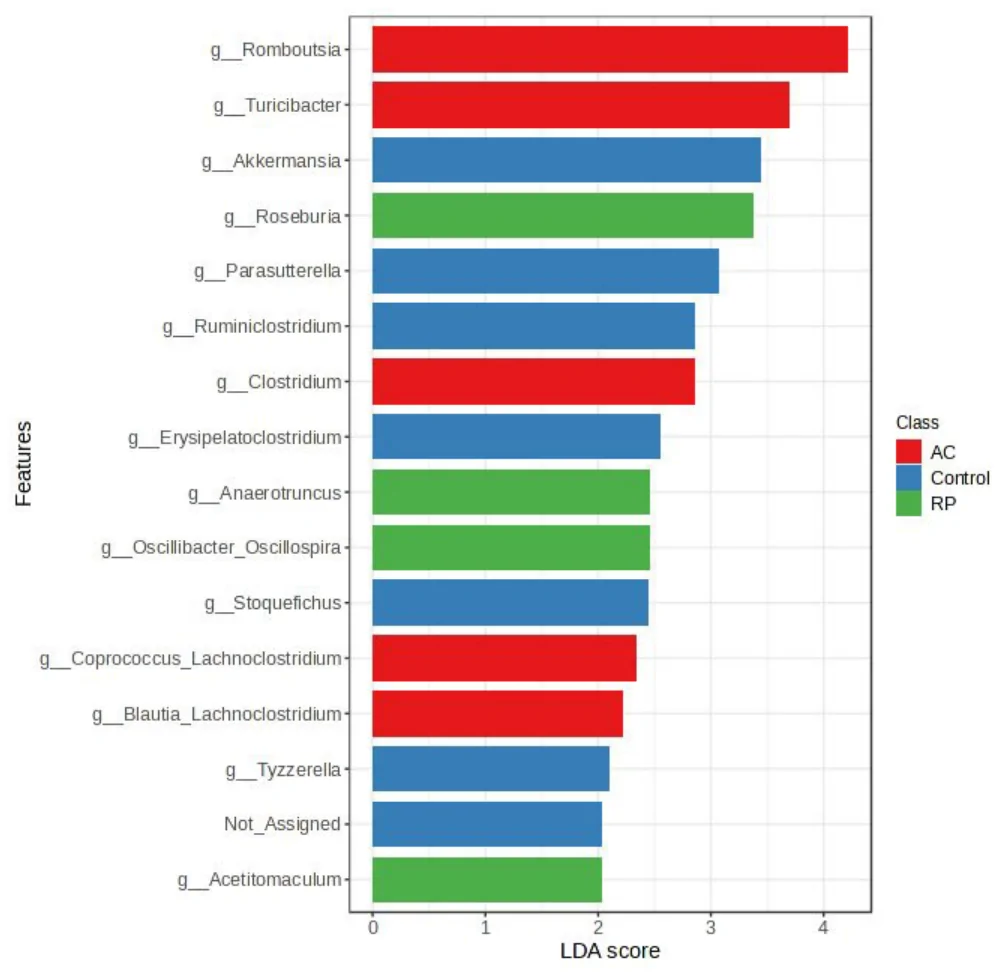
Linear discriminant analysis effect size (LEfSe) analysis identifying differentially abundant microbial taxa among groups. Horizontal bar chart showing LDA scores for taxa that differed significantly between groups, using LEfSe default settings (LDA score > 2.0; α = 0.05 for the Kruskal–Wallis and pairwise Wilcoxon tests). The analysis was performed as a single three-class comparison (control versus AC versus RP). Bars are colour-coded by group: control (blue), AC (red) and RP (green).

Linear discriminant analysis effect size (LEfSe), performed as a single three-class comparison (control vs. AC vs. RP) using default settings, identified differentially abundant taxa. The AC group was enriched in genera including *Romboutsia*, *Turicibacter*, *Clostridium*, *Coprococcus*–*Lachnoclostridium* and *Blautia*–*Lachnoclostridium*. The control group showed higher relative abundances of *Akkermansia*, *Parasutterella*, *Ruminiclostridium*, *Erysipelatoclostridium* and *Tyzzerella*. The RP group was characterised by enrichment of *Roseburia*, *Anaerotruncus*, *Oscillibacter*–*Oscillospira* and *Acetitomaculum*. The largest effect sizes were observed for *Romboutsia* and *Turicibacter* in the AC group, followed by *Akkermansia* in the control group and *Roseburia* in the RP group. Hyphenated labels denote amplicon sequence variants whose taxonomy could not be resolved unambiguously to a single genus and are distinct from the corresponding single-genus assignments (Figure 10).

## 3. Discussion

The DNBS-induced rat model is a well-established preclinical model for studying IBD, as it closely recapitulates key features of human pathology. Induced by haptenating agents such as DNBS, this model triggers a predominantly Th1-mediated immune response, leading to mucosal inflammation and tissue injury. Importantly, it mirrors the dysregulated immune environment observed in IBD, including the infiltration and activation of pathogenic Th1 and Th17 cell subsets, making it a valuable tool for investigating disease mechanisms and potential therapeutic strategies [20,21].

The immunological mechanisms underlying DNBS-induced acute colitis are characterized by a predominantly Th1 and Th17 cytokine profile [22]. A hallmark feature of this model is the pronounced alterations in inflammatory markers that serve as critical indicators of disease severity and immune activation. Previous studies have demonstrated that DNBS-induced colitis leads to significant changes in pro-inflammatory cytokines including TNF-α and IL-17, which drive the pathogenic Th1/Th17 response [23]. Additionally, anti-inflammatory cytokines such as IL-10 are markedly suppressed during acute colitis, reflecting the skewed pro-inflammatory cytokine milieu [24]. Oxidative stress markers, including MPO activity and MDA, are substantially elevated in the colonic tissue of DNBS-challenged rats, indicating increased neutrophilic infiltration and lipid peroxidation [24].

Beyond these inflammatory markers, a critical aspect of DNBS-induced colitis pathogenesis involves disruption of the intestinal epithelial barrier function. The intestinal barrier, composed of a single layer of epithelial cells joined by tight junctions, plays a crucial role in maintaining gut homeostasis by selectively regulating the passage of luminal contents while preventing the translocation of harmful substances and pathogens [25]. In DNBS-induced colitis, the integrity of this barrier is significantly compromised, as evidenced by marked downregulation of key tight junction proteins including claudin-1 and occludin [26]. Studies have shown that increased epithelial apoptosis and impaired tight junction assembly contribute to enhanced mucosal permeability, bacterial translocation, and subsequent immune activation [25]. When the intestinal tight junction barrier is compromised, it facilitates the passage of antigens and large molecules such as bacteria and their byproducts into the lamina propria, subsequently triggering an uncontrolled and dysregulated inflammatory response characteristic of IBD pathogenesis [27,28].

In the present study, these key pathological features were reproduced in our DNBS-induced colitis model. Compared with controls, DNBS-challenged rats showed significant increases in TNF-α, MPO positivity and colonic MDA, concurrent significant decreases in IL-10, and significant downregulation of occludin and claudin-1, confirming the expected pro-inflammatory, oxidative and barrier-dysfunction phenotype. Against this background, pretreatment with the two-strain formulation was associated with significantly lower TNF-α and MPO positivity and significantly higher IL-10, occludin and claudin-1 relative to untreated colitic animals, indicating that these inflammatory and barrier outcomes moved toward the control range. Serum IL-17A was unchanged across all groups. One interpretation consistent with these observations is that acute DNBS colitis in this model was predominantly Th1-driven rather than Th17-mediated; however, because IL-17A was measured in serum rather than colonic mucosa and no Th1-defining cytokines (for example IFN-γ or IL-12) were measured, this remains a hypothesis that was not directly tested here and would require mucosal immune profiling to confirm [21,22].

Two comparisons between the colitic and probiotic-treated groups did not reach statistical significance. Colonic MDA (adjusted *p* = 0.085) and the Ameho histological score (adjusted *p* = 0.310) were numerically lower in the probiotic-treated group, with moderate-to-large effect sizes (rank-biserial r = −0.750 and −0.688, respectively). A non-significant result of this kind establishes neither the absence of an effect nor that the sample size was responsible for it, and we therefore report these outcomes descriptively, with effect sizes and confidence intervals, and draw no mechanistic conclusion from them. The direction and magnitude of the MDA difference are nonetheless concordant with the significant reduction in MPO positivity in the same animals, since neutrophil-derived reactive oxygen species are a major source of colonic lipid peroxidation in this model [24]; we note this concordance as a hypothesis rather than a demonstrated link, because oxidative-pathway intermediates were not measured.

The therapeutic properties of *Lactobacillus* species in colitis have been attributed to several mechanisms in other studies, which provide context for, but were not examined in, the present work. In models using different strains and preparations, *L. rhamnosus* has been reported to modulate the inflammatory microenvironment through Toll-like receptor (TLR-2 and TLR-4) signalling, promoting regulatory T-cell differentiation and suppressing Th1/Th17 responses [29,30], to protect the epithelial barrier by preventing cytokine-induced apoptosis via the Akt/protein kinase B and p38/MAP-kinase pathways [31], and to ameliorate experimental colitis in mice through modulation of the gut microbiota [32]. In parallel, *L. paracasei* strains have been reported to enhance epithelial integrity by upregulating MUC2 and ZO-1 (strain BNCC345679) [33], to modulate the Th17/Treg balance (strain R3) [34], and, in heat-inactivated form, to attenuate lipopolysaccharide-induced epithelial injury via the TLR4/NF-κB–MLCK/MLC pathway [35]. None of these mechanistic studies examined *L. rhamnosus* L34 or *L. paracasei* B13, which are locally isolated strains, and TLR, Treg, Akt/PKB, p38/MAPK, NF-κB and MLCK/MLC signalling were not assessed here; these pathways are therefore presented as plausible mechanisms suggested by the literature rather than as demonstrated properties of the tested formulation.

Beyond these immunological and barrier-protective effects, the gut microbiota represents a critical axis in IBD pathogenesis, in which dysbiosis—reduced diversity, depletion of beneficial taxa and expansion of potentially pathogenic microorganisms—acts as both a driver and a consequence of intestinal inflammation [36]. In the present study, alpha diversity did not differ among groups, whereas beta diversity demonstrated a significant alteration in overall community structure after DNBS induction. It is important to distinguish the taxonomic differences actually observed here, which are based on 16S rRNA gene relative abundances, from the microbial functions inferred for the genera concerned, which derive from other studies and were not measured in this work (no metagenomic, metabolomic or short-chain fatty acid quantification was performed).

At the genus level, colitis was characterised by expansion of *Romboutsia* and *Turicibacter*, the two taxa with the largest LDA scores, alongside collapse of *Blautia* from a dominant population (15.51%) to 3.34%. Both *Romboutsia* and *Turicibacter* have been reported to increase in chemically induced murine colitis, and *Turicibacter* has been incorporated into microbial dysbiosis indices, consistent with their interpretation here as markers of the inflamed state rather than demonstrated drivers of inflammation [37,38]. The control group was distinguished by higher relative abundances of *Akkermansia* and *Parasutterella*. *Akkermansia muciniphila* is a mucin-degrading organism generally depleted in IBD and experimental colitis and associated in other work with a reinforced mucus layer and regulatory T-cell induction, although it is not uniformly protective and can exacerbate inflammation in some genetically susceptible hosts [39]. The probiotic-treated group showed attenuated expansion of both colitis-associated genera and was distinguished by enrichment of *Roseburia*, *Anaerotruncus*, *Oscillibacter*–*Oscillospira* and *Acetitomaculum*. *Roseburia intestinalis* is a butyrate-producing organism reported to exert anti-inflammatory and barrier-protective effects in other models [40]. Notably, *Blautia* was not re-established in probiotic-treated animals (2.25%), indicating that the formulation attenuated the expansion of inflammation-associated taxa without restoring the depleted commensal population.

These taxonomic observations are consistent with, but do not by themselves demonstrate, a microbiota-mediated contribution to the inflammatory and barrier findings, and should be read alongside three constraints. First, the probiotic-associated community did not converge toward the control community on quantitative distance-based testing, so no claim of microbial restoration or normalisation is made. Second, V3–V4 amplicon sequencing cannot resolve the administered L34 and B13 strains from resident *Lactobacillus* populations; the unchanged *Lactobacillus* abundance across groups is therefore not evidence of colonisation, and no strain-level engraftment is claimed. Third, the functional roles ascribed above to individual genera are drawn from the literature and were not measured here. Comparator studies using other *Lactobacillus* strains and preparations—for example *L. paracasei* WIS43 [41], *L. casei* IB1 [42] and *L. helveticus* LZ-R-5 [43]—report microbiota modulation accompanying reduced inflammation in DSS colitis, providing context for the present findings while differing in strain, preparation and disease model.

### Limitations

Several limitations should be considered in interpreting these findings.

First, only the two-strain combination was tested; no L34-only or B13-only arms were included. The design therefore speaks to the preparation as a whole and cannot apportion the observed effects between the constituent strains or determine whether they interact.

Second, no ethanol vehicle control group was included in the design. Because DNBS was delivered in 50% (*v*/*v*) aqueous ethanol, which can itself cause mucosal injury, the contribution of the hapten cannot be separated from that of its vehicle; the colitis phenotype described here reflects their combined effect. No probiotic-only healthy group was included, so the effects of the formulation in the absence of colitis were not determined.

Third, although the sample size was calculated prospectively and a group size of ten was planned, constraints on animal availability limited the study to eight animals per group. The realised sample size was therefore smaller than planned, and the study had less power than intended to detect differences of the magnitude assumed in the calculation. We note, however, that a non-significant result establishes neither the absence of an effect nor that the sample size was responsible for it. Accordingly, the non-significant differences in Ameho histological scores and colonic MDA are reported as numerical differences accompanied by effect sizes and confidence intervals, and we draw no conclusion from these *p*-values alone.

Fourth, mechanistic pathways were not investigated. No mucosal cytokine quantification, tissue transcript analysis, immune cell phenotyping or pathway-level analysis was performed, and IL-17A was measured in serum rather than colonic tissue. The signalling mechanisms discussed above derive from studies of other strains and preparations and are not demonstrated for the present formulation.

Fifth, we found no microbial evidence of a return toward the control community, and make no claim of restoration. Colonization and persistence of the administered organisms were not assessed; because V3–V4 amplicon data cannot discriminate L34 and B13 from resident *Lactobacillus*, genus-level abundances cannot establish engraftment. Taxonomic assignment relied on a proprietary, internally curated reference database carrying no public version identifier, which constrains independent reproducibility of the taxonomic calls.

Finally, animals were studied at a single endpoint seven days after colitis induction. The durability of the observed effects, and whether they persist after probiotic withdrawal, were not assessed.

## 4. Methods and Materials

### 4.1. DNBS Preparation

2,4-Dinitrobenzenesulfonic acid sodium salt (DNBS; 97%, catalogue number A18493, Alfa Aesar/Thermo Fisher Scientific, Waltham, MA, USA) was dissolved in 50% (*v*/*v*) aqueous ethanol to a final concentration of 140 mg/mL, giving a delivered dose of 35 mg in 0.25 mL per rat.

### 4.2. Male Sprague Dawley (SD) Rats

Twenty-four experimentally naïve male Sprague Dawley rats (6 weeks old, 180–220 g) were obtained from Nomura Siam International Co., Ltd. (Bangkok, Thailand). Animals were maintained in a strictly hygienic conventional facility in solid-bottom, open-top stainless-steel cages (36 × 75.5 × 18 cm) with wood-shaving bedding, four per cage, with the three experimental groups housed in separate cages to prevent inter-group microbial transfer by coprophagy. Environmental conditions were 23–25 °C and 55 ± 10% relative humidity under standard fluorescent lighting on a 12:12 h light/dark cycle, with *ad libitum* access to a standard chow diet (C.P. Pokphand Co., Ltd., Bangkok, Thailand) and reverse-osmosis, UV-treated water. No environmental enrichment beyond bedding was provided. Ethical approval details are given in the Institutional Review Board Statement. All procedures took place at the Laboratory Animal Facility, Faculty of Medicine, Chulalongkorn University.

### 4.3. Preparation of Experimental Colitis Rats

Acute colitis was induced with DNBS according to an established method [44]. All rats, including controls, were fasted for 24 h before instillation with free access to water; this is noted because of its relevance to the faecal microbiota analysis. Rats were lightly anaesthetised with isoflurane delivered in an induction chamber, with the vaporiser increased gradually to a maximum of 5% in oxygen at a flow rate of 1–2 L/min, and maintained at a light plane for the duration of the procedure. A polyethylene catheter was inserted rectally into the colon with the tip reaching 8 cm proximal to the anus, and the DNBS solution was instilled through the catheter (35 mg in 0.25 mL per rat). After instillation, rats were held head-down for 90 s to prevent loss of the solution. Induction was performed on two consecutive days (days 0 and 1). Because rectal instillation is a brief, non-surgical procedure performed under general anaesthesia, no post-procedural analgesia was administered; animals recovered in their home cages and were observed until fully ambulatory.

### 4.4. Probiotic Preparation

*L. rhamnosus* L34 and *L. paracasei* B13, originally obtained from infant faecal samples, were maintained as cryopreserved stocks in de Man–Rogosa–Sharpe (MRS) broth (Oxoid, Basingstoke, UK) supplemented with 20% (*v*/*v*) glycerol at −80 °C. Immediately prior to use, strains were resuscitated from frozen stocks by serial subculture (two passages) on MRS agar under strict anaerobic conditions (10% CO_2_, 10% H_2_, 80% N_2_) at 37 °C for 48 h using an anaerobic chamber with the AnaeroPack system (Thermo Scientific, Waltham, MA, USA). A single colony of each revived strain was then inoculated into MRS broth and cultured under identical conditions for 48 h. Cultures were centrifuged and resuspended in sterile 0.9% (*w*/*v*) saline, and cell density was standardised to 1.8 × 10^9^ CFU/mL by spectrophotometric determination (Bio-Rad SmartSpec Plus, Hercules, CA, USA). Viable counts were confirmed by plating serial dilutions on MRS agar. Each rat received 1 mL of suspension containing 1.8 × 10^9^ CFU/mL of each strain, yielding a combined total of 3.6 × 10^9^ CFU per animal per day.

### 4.5. Animal Protocols and Specimen Collection

**Figure 11:**
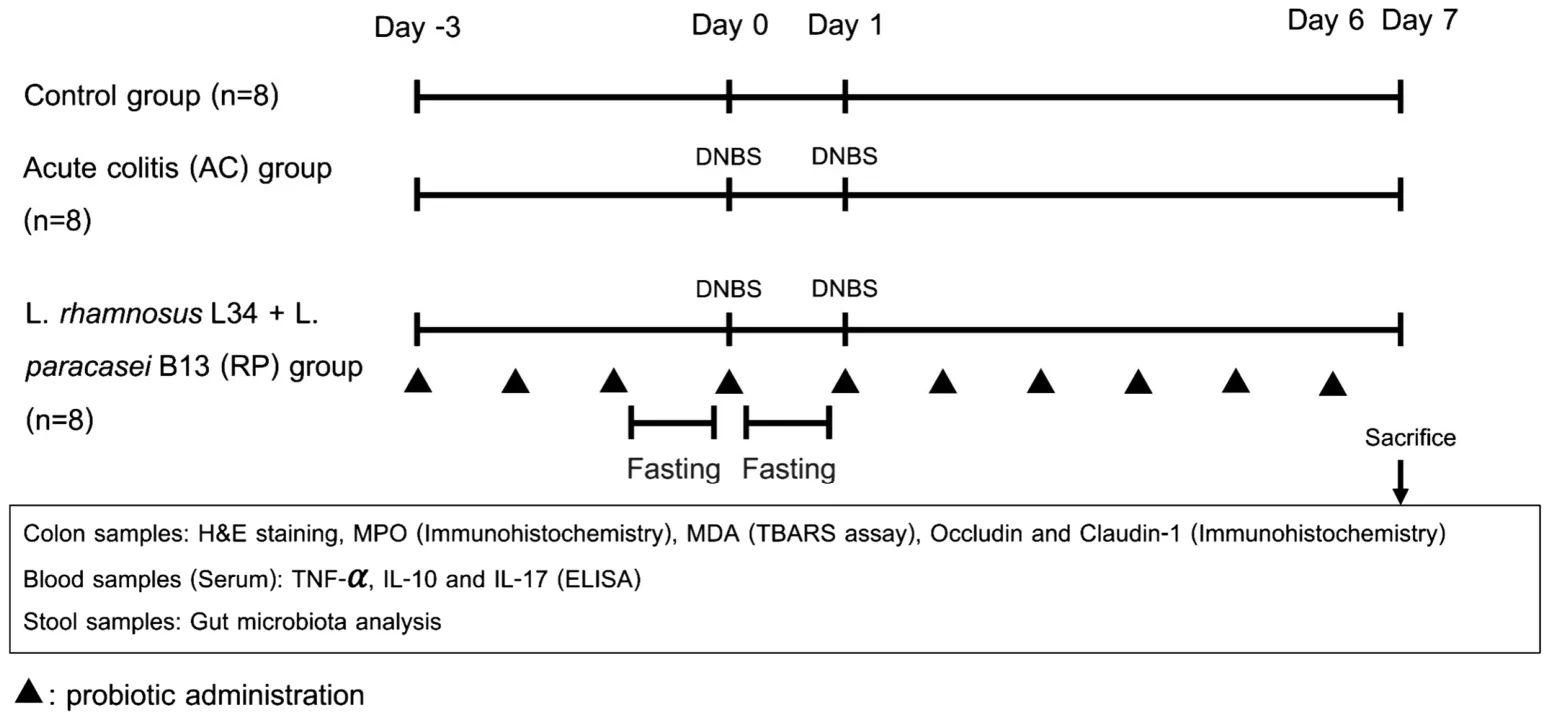
Experimental design.

After one week of acclimatization, rats were randomly allocated to three groups (*n* = 8 per group; total *n* = 24) using a computer-generated random number sequence. The experimental unit was the individual rat. The groups were as follows:

(1) Control group: Healthy rats were fed ad libitum with a standard rat chow diet.
(2) Acute colitis group (AC): Rats were given rectal DNBS solution (35 mg of DNBS in 0.25 mL of 50% (*v*/*v*) ethanol) for 2 consecutive days.
(3) Probiotics group (RP): Rats received the mixed-strain probiotic preparation (*L. rhamnosus* L34 1.8 × 10^9^ CFU/mL plus *L. paracasei* B13 1.8 × 10^9^ CFU/mL in 1 mL suspension; combined total 3.6 × 10^9^ CFU) by oral gavage once daily for 10 consecutive days, from day −3 to day 6 inclusive. Dosing was continuous throughout, with no interruption during colitis induction on days 0 and 1. Rats were sacrificed on day 7. The overall experimental design and treatment timeline are illustrated in Figure 11.

The sample size was determined prospectively. Based on IL-1β concentrations reported by Yao et al. [45] in a comparable TNBS-induced colitis model (colitis 23.05 ± 4.52 pg/mL versus probiotic-treated 16.37 ± 4.22 pg/mL), with α = 0.05 and β = 0.10, the calculated requirement was nine animals per group and the planned group size was ten. Owing to constraints on animal availability, twenty-four animals were studied, with a total of eight per group. The study was therefore conducted at a group size smaller than originally planned.

No inclusion or exclusion criteria were set a priori. All 24 rats completed the study and contributed to every analysis; no animals or data points were excluded, and *n* = 8 per group for all reported outcomes.

Rats from all groups were housed under identical conditions, cages were distributed across racks, and sample collection and assays were performed in alternating group order to minimise confounding by cage position and processing order. Group allocation was known to the investigator performing gavage and DNBS instillation. Outcome assessors were blinded: histological scoring was performed by an experienced pathologist blinded to allocation; immunohistochemical quantification was fully automated with fixed thresholds and performed by an operator blinded to allocation; and ELISA and TBARS assays were run with samples in coded order, with the operator not acting on group identity. Faecal samples were submitted to the sequencing provider under coded identifiers. To distinguish prospectively defined from retrospectively reconstructed information, the randomisation procedure, group sizes, sample-size calculation, dosing schedule and humane endpoints were specified before the experiment, whereas the detailed per-stage blinding arrangements were reconstructed during reporting from laboratory records and standard practice.

Rats were monitored daily for body weight, stool consistency, rectal bleeding and general condition. Humane endpoints were defined as greater than 20% body weight loss, severe lethargy, or persistent rectal bleeding. No animal reached a humane endpoint, and no unexpected adverse events or deaths occurred during the study. The primary outcomes were changes in inflammatory markers and tight junction proteins; faecal microbiota composition was a secondary outcome.

At the end of the study (day 7), fresh faeces were collected for gut microbiota analysis. Rats were then deeply anaesthetised by intraperitoneal injection of thiopental sodium (100 mg/kg body weight), and the rats were euthanised by exsanguination via cardiac puncture, which also provided the blood samples for serum analysis. The abdominal wall was opened and the whole colon was rapidly removed and washed with cold normal saline. Part of the colon was freeze-dried and stored at −70 °C until analysis; other portions were fixed in 10% formalin for histological examination. Blood was allowed to clot at room temperature for 2 h and centrifuged at 1000× *g* for 20 min, and serum was collected for further evaluation.

### 4.6. Colon Histopathology

The colonic tissue was transferred into a fixative of 10% formalin solution immediately after collection at room temperature. The colonic tissue was embedded in paraffin, sectioned at 5 μm. Briefly, the sections were deparaffinised with xylene, rehydrated with graded alcohols and stained with haematoxylin and eosin for light-microscopic evaluation. An experienced pathologist evaluated all samples while being blinded to the experiment. Histopathologic lesions of colon samples were examined across all fields and graded as shown in Table 1 [46].

### 4.7. Serum Analysis

Serum concentrations of TNF-α, IL-10 and IL-17A were determined by quantitative sandwich ELISA. TNF-α was measured using the Rat TNF-α Quantikine ELISA kit (catalogue RTA00, R&D Systems, Minneapolis, MN, USA; assay range 12.5–800 pg/mL, minimum detectable dose < 5.0 pg/mL), with serum assayed at a 1:40 dilution in the supplied calibrator diluent. IL-10 was measured using the Rat IL-10 Quantikine ELISA kit (catalogue R1000, R&D Systems; assay range 31.2–2000 pg/mL, sensitivity 10 pg/mL), assayed undiluted. IL-17A was measured using the Rat IL-17/IL-17A ELISA kit (catalogue NBP1-92705, Novus Biologicals, Centennial, CO, USA; assay range 1.56–100 pg/mL, limit of detection 1.0 pg/mL), assayed at a 1:4 dilution. All samples were assayed in duplicate, and reported concentrations incorporate the respective dilution factors. All measured values fell within the calibration range of their respective assays; no value required extrapolation beyond the standard curve or re-assay at a different dilution. Absorbance was read at 450 nm and concentrations were interpolated from the standard curves.

### 4.8. Malondialdehyde (MDA) Level by Thiobarbituric Acid Reactive Substances (TBARS) Assay

Colonic lipid peroxidation was assessed by measuring malondialdehyde (MDA) levels using the thiobarbituric acid reactive substances (TBARS) assay. Briefly, colonic tissue (0.1 g) was homogenised on ice for 30 min in 1 mL of RIPA buffer (Cell Signaling Technology, Danvers, MA, USA) containing protease inhibitor cocktail (Sigma-Aldrich, Darmstadt, Germany). Protein concentration in the resulting supernatant was quantified by bicinchoninic acid (BCA) assay (Pierce, Thermo Scientific, Rockford, IL, USA).

MDA levels were subsequently determined using a commercial TBARS assay kit (Cayman Chemical, Ann Arbor, MI, USA). The supernatant was mixed with a reagent solution containing 20% acetic acid, 0.8% thiobarbituric acid, and 8.1% sodium dodecyl sulphate, then heated at 95 °C for 1 h and centrifuged at 1600× *g* for 10 min. Absorbance of the supernatant was measured at 532 nm and MDA concentrations were expressed as nmol/mg protein.

### 4.9. Immunohistochemistry

Colonic expression of myeloperoxidase (MPO) and the tight junction proteins occludin and claudin-1 was assessed by immunohistochemistry (IHC) using a tissue microarray (TMA) approach. Paraffin-embedded donor blocks were retrieved, sectioned, and stained with haematoxylin and eosin (H&E). An experienced pathologist identified and marked representative areas on each slide, and the corresponding regions in the donor blocks were cored four to six times. Cores were transferred into a recipient TMA block for subsequent analyses.

The TMA block was sectioned at 5 µm, deparaffinised in xylene and graded alcohols, and rehydrated with wash buffer. For MPO detection, sections were incubated overnight at 4 °C with a goat polyclonal anti-MPO primary antibody (1:500; R&D Systems, USA), followed by a donkey anti-goat IgG (H&L)–HRP secondary antibody (1:500; Abcam, Cambridge, UK) for 60 min at room temperature. Slides were then incubated with high-sensitivity streptavidin-HRP conjugate (HSS-HRP) for 30 min and developed with DAB chromogen solution for 15 min.

For occludin and claudin-1, sections were incubated at room temperature for 1 h with rabbit polyclonal antibodies against occludin (1:500; Novus Biologicals, USA) and claudin-1 (1:200; Novus Biologicals, USA), followed by overnight incubation at 4 °C in a humidified chamber. A goat anti-rabbit IgG (H&L)–HRP secondary antibody (1:1000; Abcam, UK) was applied for 1 h at room temperature.

All slides, including haematoxylin and eosin-stained sections and immunohistochemical preparations, were digitised using the Aperio ScanScope whole-slide scanner (Aperio Technology, Vista, CA, USA) at 40× objective magnification with the scanner’s integrated camera, and images were viewed and exported using Aperio ImageScope software (version 12.3.2.8013). Positive pixel count analysis was performed with appropriate positive and negative tissue controls, using the default hue and intensity threshold settings of the Aperio algorithm. The same threshold settings were applied to all slides and all markers, without slide-specific adjustment, and analysis was performed automatically in batch by an operator blinded to group allocation. Staining intensity was classified by the algorithm as negative, weakly positive, positive or strongly positive. For each animal, images from all four to six cores were combined and analysed together, and the outcome was expressed as the area fraction of positive staining, defined as the sum of positive and strongly positive pixels divided by the total number of counted pixels. This yields a dimensionless proportion between 0 and 1 and represents staining positivity rather than protein concentration or, in the case of MPO, enzymatic activity. A single area fraction was obtained per animal per marker, and the animal was the experimental unit for all statistical comparisons (*n* = 8 per group). Group-level values are reported as medians with interquartile ranges.

### 4.10. Faecal Microbiota

Faecal specimens were collected from all rats at the experimental endpoint and stored at −80 °C pending analysis. Microbial DNA was extracted using the ZymoBIOMICS-96 MagBead DNA Kit (Zymo Research, Irvine, CA, USA) on an automated platform according to the manufacturer’s instructions. Process controls were included: the ZymoBIOMICS Microbial Community Standard served as a positive extraction control and a blank extraction as the negative control. Quantitative PCR of the negative control yielded 27 16S rRNA gene copies/µL (Ct 34.45), compared with 6.81 × 10^9^ copies/µL for the positive control, indicating negligible reagent and process contamination. The V3–V4 hypervariable regions of the bacterial 16S rRNA gene were amplified and processed using the ZymoBIOMICS Targeted Metagenomic Sequencing Service (Zymo Research).

### 4.11. Microbiota Characterization and Sequencing Analysis

Faecal bacterial communities were profiled by 16S rRNA gene sequencing targeting the V3–V4 hypervariable regions. Libraries were constructed using the Quick-16S NGS Library Prep Kit (Zymo Research, Irvine, CA, USA) with proprietary custom V3–V4 primers, in which PCR was performed in a real-time thermocycler to limit chimera formation. Products were quantified by qPCR, pooled at equal molarity, cleaned with the Select-a-Size DNA Clean & Concentrator (Zymo Research), and quantified by TapeStation (Agilent Technologies, Santa Clara, CA, USA) and Qubit (Thermo Fisher Scientific, Waltham, MA, USA). The final library was sequenced on an Illumina NextSeq 2000 (Illumina, San Diego, CA, USA) with a P1 reagent kit (600 cycles) and a 30% PhiX spike-in, generating paired-end reads of 321 bp (read 1) and 281 bp (read 2). Amplicon sequence variants (ASVs) were inferred using the DADA2 pipeline [47], including quality filtering and removal of chimeric sequences. Taxonomic assignment was performed using UCLUST within QIIME v1.9.1 [48] against the ZymoBIOMICS internally curated 16S reference database. Total sequencing depth was 7,073,318 read pairs (median 289,494 per sample, range 223,266–406,693), which did not differ among groups (Kruskal–Wallis *p* = 0.564). Final ASV counts ranged from 222 to 461 per sample (median 312). Per-sample read processing and ASV counts are provided in Supplementary Table S1, and a group-level summary of read processing in Supplementary Table S2.

### 4.12. Diversity and Differential Abundance Analysis

Microbiota diversity was assessed using alpha and beta diversity metrics. Alpha diversity (Chao1, Shannon, Simpson and observed ASVs) was computed on data rarefied to 20,000 sequences per sample, averaged across 10 iterations, and compared among groups by Kruskal–Wallis test. Beta diversity was assessed by principal coordinate analysis of Bray–Curtis dissimilarities. Differences in community composition were tested by permutational multivariate analysis of variance (PERMANOVA) [49] and homogeneity of multivariate dispersion by PERMDISP [50], both implemented in scikit-bio v0.7.4 under Python 3.13 with 9999 unrestricted permutations of group labels and a fixed random seed; pairwise comparisons were Bonferroni-corrected for three tests. Because heterogeneous dispersion can influence PERMANOVA in unbalanced designs but not in balanced ones [51], and the present design was balanced (*n* = 8 per group), both tests are reported together to distinguish location from dispersion effects. To test whether the probiotic-treated community was closer to the control state, the mean Bray–Curtis distance from each animal to the eight control animals was compared between the AC and RP groups using a permutation test with 99,999 permutations of group labels, which accounts for the dependence structure of the pairwise distances. Differentially abundant taxa were identified by linear discriminant analysis effect size (LEfSe) [52] using default settings (LDA score > 2.0; α = 0.05 for the Kruskal–Wallis and pairwise Wilcoxon tests), performed as three pairwise group comparisons.

### 4.13. Statistical Analysis

Normality of continuous variables was assessed using the Shapiro–Wilk test. Because several outcomes deviated from normality, all outcomes are reported as medians with interquartile ranges, the latter computed using the exclusive quartile method (equivalent to R quantile type 6). Between-group comparisons for all outcomes were performed using the Kruskal–Wallis test, followed by Dunn’s post hoc test with Bonferroni correction applied once across the three pairwise comparisons within each outcome; no correction was applied across outcomes. Adjusted *p*-values reported as 1.000 are Bonferroni-adjusted values truncated at unity and indicate the absence of a detectable difference rather than equivalence. Effect sizes are reported as epsilon-squared for each Kruskal–Wallis test and as Hodges–Lehmann median differences with 95% confidence intervals together with rank-biserial correlations for each pairwise comparison; confidence intervals are not adjusted for multiplicity, so a confidence interval may exclude zero while the corresponding adjusted *p*-value exceeds 0.05, in which case the adjusted *p*-value should be used for inference. Full effect-size results are given in Supplementary Tables S3 and S4. Statistical significance was set at *p* < 0.05. Analyses were performed in SPSS version 29.0.1 (IBM Corp., Armonk, NY, USA) and verified in Python 3.13 using SciPy v1.18.1 and scikit-posthocs. In all figures, numbers above comparison brackets denote Bonferroni-adjusted p-values from Dunn’s post hoc test. In all box plots, the centre line shows the median, the box the interquartile range and the whiskers 1.5 × IQR; points beyond the whiskers are shown individually as outliers.

## 5. Conclusions

Preventive administration of a two-strain formulation of *L. rhamnosus* L34 and *L. paracasei* B13 was associated with reduced inflammatory and oxidative markers and better-preserved epithelial junction protein staining in rats with DNBS-induced colitis. Against untreated colitic animals, treated rats showed lower serum TNF-α and colonic MPO positivity together with higher IL-10, occludin and claudin-1 positivity; colonic MDA and histological scores trended in the same direction without reaching significance, and serum IL-17A was unaffected. The faecal community of treated animals was distinct from those of both comparison groups, yet distance-based testing gave no indication that it had moved toward the control state. These results characterise what this particular two-strain preparation did in a rat model of DNBS-induced colitis. Whether the strains act independently, additively or in concert was outside the scope of this design and conclusions in this area await studies with single-strain comparison groups, as does identification of the mechanisms responsible for the effects observed.

## Figures and Tables

**Table 1:** Ameho’s score for histopathological colitis grading.

Grade of Colitis	Microscopic Findings
0	Histological findings identical to normal rats.
1	Mild mucosal and/or submucosal inflammatory infiltrate (admixture of neutrophils) and oedema. Punctate mucosal erosions often associated with capillary proliferation. Muscularis mucosae intact.
2	Grade 1 changes involving more than 50% of the specimen.
3	Prominent inflammatory infiltrate and oedema (neutrophils usually predominating) frequently with deeper areas of ulceration extending through the muscularis mucosae into the submucosa. Rare inflammatory cells invading the muscularis propriae but without muscle necrosis.
4	Grade 3 changes involving more than 50% of the specimen.
5	Extensive ulceration with coagulative necrosis bordered inferiorly by numerous neutrophils and lesser numbers of mononuclear cells. Necrosis extends deeply into the muscularis propria.
6	Grade 5 changes involving more than 50% of the specimen.

## Data Availability

The raw 16S rRNA gene sequencing reads generated in this study have been deposited in the NCBI Sequence Read Archive under BioProject accession PRJNA1548177. All other data supporting the findings of this study are available from the corresponding author upon reasonable request.

## Supplementary Materials

The following supporting information can be downloaded at https://www.mdpi.com/article/10.3390/ijms27219961/s1.
