# Supplementary material for: Effects of a Two-Strain Lactobacillus Formulation (Lactobacillus rhamnosus L34 and Lactobacillus paracasei B13) on Inflammatory and Oxidative Markers, Epithelial Junction Proteins, and Fecal Microbiota in Rats with DNBS-Induced Colitis
Source: Int J Mol Sci. 2026 Nov 6;27(21):9961. doi: 10.3390/ijms27219961 (PMC13764108; doi:10.3390/ijms27219961)
Supplement: Supplementary file 1 [file ijms-27-09961-s001.zip › TableS4_effect_sizes.pdf]

Table S4 effect sizes

Outcome	Comparison	Unit	Hodges-Lehmann differenc	95% CI	Rank-biserial r	Dunn p (Bonferroni)
**Ameho histological score**	AC vs Control	(score)	5	1 to 5	1	<0.0001
**Ameho histological score**	RP vs AC	(score)	-4	-4 to 0	-0.688	0.3096
**Ameho histological score**	RP vs Control	(score)	1	1 to 3	1	0.0184
**Serum TNF-α**	AC vs Control	pg/mL	9,260	3,880 to 14,160	1	0.0018
**Serum TNF-α**	RP vs AC	pg/mL	-9,235	-14,240 to -4,210	-1	0.0023
**Serum TNF-α**	RP vs Control	pg/mL	40	-950 to 1,220	0.031	1
**Serum IL-10**	AC vs Control	pg/mL	-161	-351 to -52.0	-0.812	0.0362
**Serum IL-10**	RP vs AC	pg/mL	259	187 to 367	1	0.0008
**Serum IL-10**	RP vs Control	pg/mL	122	-85.0 to 259	0.406	0.7737
**Serum IL-17**	AC vs Control	pg/mL	4	-32.0 to 32.0	0.156	1
**Serum IL-17**	RP vs AC	pg/mL	-2.5	-58.0 to 7.0	-0.125	1
**Serum IL-17**	RP vs Control	pg/mL	-0.5	-36.0 to 8.0	-0.016	1
**Colonic MDA**	AC vs Control	nmol/mg protein	14.46	8.37 to 20.60	0.906	0.0018
**Colonic MDA**	RP vs AC	nmol/mg protein	-11.94	-18.17 to -4.65	-0.75	0.0851
**Colonic MDA**	RP vs Control	nmol/mg protein	2.1	-0.79 to 6.13	0.469	0.6478
**Colonic MPO positivity**	AC vs Control	area fraction	0.1535	0.0420 to 0.2360	1	0.0276
**Colonic MPO positivity**	RP vs AC	area fraction	-0.159	-0.2390 to -0.0480	-1	<0.0001
**Colonic MPO positivity**	RP vs Control	area fraction	-0.005	-0.0130 to -0.0010	-0.703	0.3325
**Colonic occludin positivity**	AC vs Control	area fraction	-0.08	-0.1100 to -0.0400	-0.984	0.0008
**Colonic occludin positivity**	RP vs AC	area fraction	0.06	0.0300 to 0.0900	0.922	0.0136
**Colonic occludin positivity**	RP vs Control	area fraction	-0.02	-0.0500 to 0.0200	-0.328	1
**Colonic claudin-1 positivit**	AC vs Control	area fraction	-0.14	-0.2400 to -0.0900	-1	0.0052
**Colonic claudin-1 positivit**	RP vs AC	area fraction	0.17	0.1000 to 0.2600	1	0.0007
**Colonic claudin-1 positivit**	RP vs Control	area fraction	0.02	-0.0200 to 0.0700	0.234	1
