# Supplementary material for: Effects of a Two-Strain Lactobacillus Formulation (Lactobacillus rhamnosus L34 and Lactobacillus paracasei B13) on Inflammatory and Oxidative Markers, Epithelial Junction Proteins, and Fecal Microbiota in Rats with DNBS-Induced Colitis
Source: Int J Mol Sci. 2026 Nov 6;27(21):9961. doi: 10.3390/ijms27219961 (PMC13764108; doi:10.3390/ijms27219961)
Supplement: Supplementary file 1 [file ijms-27-09961-s001.zip › TableS1_sequencing_summary.pdf]

Table S1 sequencing summary

internal_id	customer_label	group	rawseqs(R1+R2)	trimmed_seqs(R1+R2	dada2_infered	chimera_seqs	chimera_free_seqs	seqs(after_size_filtration	final_unique_seqs
**zr15904_1V3V4**	C1	Control	574182	481308	230994	24603	206391	204970	461
**zr15904_2V3V4**	C2	Control	813386	682080	332323	56423	275900	274460	320
**zr15904_3V3V4**	C3	Control	640164	539710	260598	34596	226002	224470	401
**zr15904_4V3V4**	C6	Control	543612	455746	222049	32157	189892	189036	265
**zr15904_5V3V4**	C7	Control	526528	443424	217772	32456	185316	184531	222
**zr15904_6V3V4**	C8	Control	608674	510834	249665	38020	211645	210595	229
**zr15904_7V3V4**	C9	Control	599770	501844	243527	34656	208871	207548	310
**zr15904_8V3V4**	C10	Control	583792	487892	235875	35992	199883	198615	324
**zr15904_9V3V4**	AC1	AC	648910	543112	265246	39643	225603	224365	320
**zr15904_10V3V4**	AC2	AC	516524	434508	210888	25524	185364	184374	306
**zr15904_11V3V4**	AC4	AC	550702	462684	225594	35230	190364	189315	276
**zr15904_12V3V4**	AC5	AC	499108	418250	201889	26831	175058	173633	373
**zr15904_13V3V4**	AC6	AC	519958	437834	212887	22498	190389	189327	288
**zr15904_14V3V4**	AC7	AC	496020	417442	202304	25664	176640	175616	313
**zr15904_15V3V4**	AC8	AC	662552	555726	270820	50917	219903	218682	264
**zr15904_16V3V4**	AC10	AC	624400	525752	254818	39827	214991	213636	311
**zr15904_17V3V4**	RP2	RP	446532	375210	181106	27866	153240	151923	306
**zr15904_18V3V4**	RP4	RP	496484	417308	200444	35059	165385	164054	362
**zr15904_19V3V4**	RP5	RP	541702	454196	220178	30933	189245	187958	313
**zr15904_20V3V4**	RP6	RP	551634	463452	225525	30436	195089	194224	284
**zr15904_21V3V4**	RP7	RP	729908	612948	298076	36321	261755	260271	347
**zr15904_22V3V4**	RP8	RP	629178	528298	255030	44916	210114	208655	396
**zr15904_23V3V4**	RP9	RP	687458	577048	281126	55621	225505	224492	279
**zr15904_24V3V4**	RP10	RP	655458	551222	268958	31716	237242	235862	341
