# Supplementary material for: Effects of a Two-Strain Lactobacillus Formulation (Lactobacillus rhamnosus L34 and Lactobacillus paracasei B13) on Inflammatory and Oxidative Markers, Epithelial Junction Proteins, and Fecal Microbiota in Rats with DNBS-Induced Colitis
Source: Int J Mol Sci. 2026 Nov 6;27(21):9961. doi: 10.3390/ijms27219961 (PMC13764108; doi:10.3390/ijms27219961)
Supplement: Supplementary file 1 [file ijms-27-09961-s001.zip › TableS2_group_summary.pdf]

Table S2 group summary

Metric	Control median [IQR]	AC median [IQR]	RP median [IQR]	Overall median [range]
**Raw reads (R1+R2)**	591,781 [551,254-632,292	535,330 [503,462-642,782	590,406 [507,788-679,458	578,987 [446,532-813,386]
**After trimming**	494,868 [462,136-532,491	450,259 [422,314-538,772	495,875 [426,530-570,592	484,600 [375,210-682,080]
**DADA2-inferred**	239,701 [224,285-257,865	219,240 [204,450-262,639	240,278 [205,378-278,084	233,434 [181,106-332,323]
**Chimeric**	34,626 [32,232-37,513]	31,030 [25,559-39,781]	33,388 [30,560-42,767]	34,626 [22,498-56,423]
**Chimera-free**	207,631 [192,390-222,413	190,376 [178,821-218,675	202,602 [171,350-234,308	203,137 [153,240-275,900]
**After size filtration**	206,259 [191,431-221,001	189,321 [177,806-217,420	201,440 [170,030-233,020	201,792 [151,923-274,460]
**Final ASVs**	315 [238-382]	308 [279-318]	327 [290-358]	312 [222-461]
