# Supplementary material for: Effects of a Two-Strain Lactobacillus Formulation (Lactobacillus rhamnosus L34 and Lactobacillus paracasei B13) on Inflammatory and Oxidative Markers, Epithelial Junction Proteins, and Fecal Microbiota in Rats with DNBS-Induced Colitis
Source: Int J Mol Sci. 2026 Nov 6;27(21):9961. doi: 10.3390/ijms27219961 (PMC13764108; doi:10.3390/ijms27219961)
Supplement: Supplementary file 1 [file ijms-27-09961-s001.zip › TableS3_descriptives_KW.pdf]

Table S3 descriptives KW

Outcome	Unit	Control	AC	RP	Kruskal-Wallis H (df=2	p	epsilon-squared
**Ameho histological score**	(score)	0 [0–0]	5 [2–5]	1 [1–2.5]	19.51	<0.0001	0.834
**Serum TNF-α**	pg/mL	2,745 [1,815–3,305]	11,800 [6,922–16,625]	2,610 [1,778–3,458]	15.365	0.0005	0.636
**Serum IL-10**	pg/mL	277 [192–492]	140 [83.3–198]	418 [316–460]	13.895	0.001	0.566
**Serum IL-17**	pg/mL	121 [119–159]	127 [119–173]	126 [119–129]	0.304	0.8588	-0.081
**Colonic MDA**	nmol/mg protein	2.56 [1.24–5.37]	17.98 [11.50–23.84]	3.96 [2.79–8.89]	12.065	0.0024	0.479
**Colonic MPO positivity**	area fraction	0.0305 [0.0283–0.0372	0.1850 [0.0745–0.2652	0.0260 [0.0222–0.0290	17.969	0.0001	0.76
**Colonic occludin positivity**	area fraction	0.9450 [0.9025–0.9600	0.8650 [0.8350–0.8800	0.9200 [0.8950–0.9475	14.717	0.0006	0.606
**Colonic claudin-1 positivit**	area fraction	0.4750 [0.4450–0.5125	0.3400 [0.2375–0.3875	0.4950 [0.4700–0.5325	15.689	0.0004	0.652
